# Impact of the COVID-19 pandemic on the work of clinical psychologists in Austria: results of a mixed-methods study

**DOI:** 10.3389/fpsyg.2024.1302442

**Published:** 2024-04-25

**Authors:** Paola Santillan-Ramos, Elke Humer, Yvonne Schaffler, Christoph Pieh, Thomas Probst, Anna Felnhofer, Oswald Kothgassner, Ingeborg Netzer, Andrea Jesser

**Affiliations:** ^1^Department for Psychosomatic Medicine and Psychotherapy, University of Continuing Education Krems, Krems an der Donau, Austria; ^2^Faculty of Psychotherapy Science, Sigmund Freud University, Vienna, Austria; ^3^Division of Psychotherapy, Department of Psychology, Paris Lodron University of Salzburg, Salzburg, Austria; ^4^Division of Pediatric Pulmonology, Allergology and Endocrinology, Department of Pediatrics and Adolescent Medicine, Medical University of Vienna, Vienna, Austria; ^5^Department of Child and Adolescent Psychiatry, Comprehensive Center for Pediatrics, Medical University of Vienna, Vienna, Austria; ^6^Österreichischer Arbeitskreis für Gruppentherapie und Gruppendynamik, Fachsektion Integrative Gestalt Therapy Vienna, Vienna, Austria

**Keywords:** COVID-19, clinical psychologist, mixed-methods study, mental health professionals, public health

## Abstract

**Introduction:**

Clinical psychologists in Austria shouldered a large part of the massive increase in demand for mental health services caused by the COVID-19 pandemic. This study aimed to find out how the pandemic affected their work and to gather information on how best to support the profession in the event of a crisis.

**Methods:**

*N* = 172 Austrian clinical psychologists participated in a cross-sectional online survey between 11 April 2022 and 31 May 2022, including both closed and open-ended questions about their work. Open-ended questions were analyzed using qualitative content analysis. A mixed-methods analysis was conducted to test correlations between the categories derived from the qualitative analysis and professional variables.

**Results:**

The analyses revealed that clinical psychologists, especially those with more years of experience, perceived an increased need for clinical psychological treatment, especially for children and adolescents, a lack of coverage for clinical psychological treatment by health insurance, a change to remote treatment formats, and a number of burdens associated with complying with COVID-19 measures.

**Discussion:**

Clinical psychologists reported an urgent need to increase resources in both outpatient and inpatient settings and to promote health insurance coverage. To support the clinical psychology profession in providing high-quality work in times of crisis, there is a need to facilitate more opportunities for team and peer exchange, as well as financial support in the event of loss of income.

## 1 Introduction

SARS-CoV-2 (severe acute respiratory syndrome coronavirus type 2) is a new coronavirus identified in early 2020 as the causative agent of COVID-19. The main transmission route of SARS-CoV-2 is the respiratory ingestion of virus-containing particles produced by breathing, coughing, talking, singing, and sneezing (Wang et al., 2021). In March 2020, the World Health Organization (WHO) declared the infection a pandemic due to the high number of cases, the many deaths, and the large number of countries affected. At the WHO press conference on 11 March 2020, the following points were identified as important regarding COVID-19: Prevention, preparedness, public health, and political leadership. Particular emphasis was placed on the call for the general population to protect each other from the virus (World Health Organization, 2020).

Between March and May 2020, the Austrian government declared restrictive measures that limited freedom and social life to reduce the risk of new infections in the population and, at the same time, protected the health system from possible overload (Pollak et al., 2020). In addition to preventive hygiene measures such as washing hands or sneezing into the crook of the arm, the Austrian government imposed a lockdown with only a few exceptions: Shopping for food or medicine, helping others, going to work, or going for a walk. Individuals were called upon to keep a distance of 2 m from others. Meeting other people who did not live in the same household was generally forbidden. The curfew also meant the closure of shops, schools, and cultural institutions.

With the introduction of the COVID-19 vaccination in Austria in December 2020 and declining infection rates between May and July 2021, COVID-19 restrictions could be relaxed. However, in late summer and fall of 2021, a new variant of the virus spread. Infection numbers rose rapidly, reaching a record high in November 2021 (Pollak et al., 2021). ICU bed occupancy also increased sharply, prompting the federal government to impose another lockdown until January 2022. In February 2022, compulsory vaccination officially went into effect (Walcherberger et al., 2023). Despite high infection numbers, COVID-19 measures for the general population in Austria were relaxed in March 2022 (Walcherberger et al., 2023).

Research has shown that the pandemic and the measures taken to contain the virus have had a negative impact on mental health worldwide. Increases in the prevalence of symptoms of depression, anxiety, insomnia, stress, substance abuse, and eating disorders have been reported (Roberts et al., 2021; Sideli et al., 2021; Mahmud et al., 2023). Fears of illness, reduced social contact, and financial concerns have been identified as underlying factors in mental health deterioration (Xiong et al., 2020). Feelings of loneliness due to self-isolation have often been cited as a cause of mental health problems (Killgore et al., 2020) and are positively correlated with symptoms of anxiety, depression, and stress (Gu et al., 2021).

In Austria, a study conducted early in the pandemic showed an increase in symptoms of depression, anxiety, and insomnia compared with pre-COVID-19 levels (Pieh et al., 2020). A follow-up study showed that the adverse health effects of the COVID-19 pandemic persisted for several months after the outbreak and the end of the lockdown measures (Pieh et al., 2021). In April 2022, the mental health burden was still high, with rates of depressive symptoms continuing to rise (Humer et al., 2022). Younger adults (<35 years old) and people with a low income (<€ 2,000 net household income per month) were particularly affected by the deterioration in mental health (Humer et al., 2022). Austria is not an isolated case. Studies conducted in countries with high COVID-19 cases and death rates, such as Italy, Spain, the United States and the UK, have shown an increase in depression, anxiety, insomnia and PTSD symptoms among the general population (Budimir et al., 2021). A comparison of severe mental health symptoms in the UK and Austria during the COVID-19 lockdown revealed a higher prevalence of severe depression, anxiety, and insomnia symptoms in the UK than in Austria (Budimir et al., 2021). Vulnerability factors that influence the impact of the COVID-19 pandemic on the mental health of populations were identified in both British and Austrian populations. Signs of vulnerability encompass poverty, existing mental health conditions, and inadequate social support. Women, children, young adults (aged 18–30), the unemployed, and individuals living alone appear to be the most affected groups (Li and Wang, 2020; Simon et al., 2021).

Mental health services are urgently needed to be able to professionally counter the negative consequences of the pandemic on the mental health of the population in Austria. Clinical psychological treatment exists on an equal footing with other curative procedures for treating mental disorders and states of suffering, such as psychiatric treatment or psychotherapy. While health insurance funds reimburse all or part of the costs of psychotherapy or psychiatric treatment, clinical psychological treatment and diagnostics costs are covered only in the institutional sector (Berufsverband Österreicher PsychologInnen, 2023). Individuals seeking to become clinical psychologists in Austria, must first complete a university degree in psychology, which entails 300 ECTS credits. After finishing the 5-year master’s program in psychology, aspiring clinicians must then undertake 1–2 years of postgraduate specialized training in both theoretical approaches and practical techniques. When all these requirements have been fulfilled, then registration on the Ministry’s list of clinical psychologists is completed and the license to practice as a clinical psychologist is granted (Berufsverband Österreicher PsychologInnen, 2023). Health insurance providers finance clinical psychological services like assessment and therapy when rendered in institutional settings. However, in private practice, only diagnostic evaluations are covered, while treatment and counseling interventions must be paid for out-of-pocket by patients. No subsidies or cost coverage exist from health insurance for clinical psychologists’ private therapeutic practice. Only in the case of acute stress associated with psychological trauma, up to 10 h of clinical psychological crisis support and treatment are covered by the Federal Social Office according to the Crime Victims Act (Öffentliches Gesundheitsportal Österreichs, 2019; Bundeministerium für Soziales, Gesundheit, Pflege und Konsumentenschutz, 2023). Ongoing negotiations are being conducted between the professional body representing clinical psychologists and the primary governing bodies overseeing Austrian social insurance institutions, along with the Federal Ministry of Health. The objective of these negotiations is to integrate clinical psychological treatment into the standard care framework, thereby recognizing it as a covered service under the statutory health and social insurance schemes. This development aims to establish clinical psychological interventions as a mandated benefit, whereby individuals insured under the public health and social security systems would have access to such therapeutic services as an entitlement, without incurring additional out-of-pocket expenses (Berufsverband Österreicher PsychologInnen, 2023).

The measures used to contain the SARS-CoV-2 have massively changed the work and the working conditions in the health and social sectors. Although face-to-face treatments were still permitted in Austria despite lockdown measures, the practice of clinical psychology in Austria was nonetheless affected by the lockdown measures. In May 2020, the Professional Association of Austrian Psychologists published the COVID-19 Guidelines and Fact Sheet, which contained recommendations for clinical psychological work in independent practice (Berufsverband Österreicher PsychologInnen, 2020). In addition to appropriate hygiene or cleaning and disinfection measures, such as paying attention to hand hygiene (no shaking, regular hand washing and/or disinfection) or wearing protective masks, the Federal Ministry of Social Affairs, Health and Consumer Protection also recommended that consultations and treatments be carried out via the internet and/or telephone for patients at high health risk (Berufsverband Österreicher PsychologInnen, 2020). The decision of whether the use of online therapy instead of face-to-face contact would also be suitable in other cases was left to the practitioner (Berufsverband Österreicher PsychologInnen, 2020). The COVID-19 measures in the health services sector remained in place in 2022 despite the relaxation of COVID-19 measures within the general population (Berufsverband Österreicher PsychologInnen, 2020).

As the health care system in general, and the mental health care system in particular, faced significant challenges due to the pandemic, it is important to investigate how clinical psychologists experienced the changes and challenges in carrying out their professional work. To provide quality clinical psychological treatment, it is essential to examine the conditions of this treatment and the burdens and needs of those providing the treatment. For this reason, the present study collected data on the psychological well-being of clinical psychologists during the pandemic and investigated which factors they found stressful and what helped them to cope with stress. It also looked at how clinical psychologists perceived changes in their work and what support they needed. Some of the results have already been published. Humer et al. (2023) showed that clinical psychologists had better mental health than the general population during the COVID-19 pandemic, suggesting that they experienced less stress or had more coping strategies to deal with stress. This question was addressed in another publication based on this study by Jesser et al. (2024), which looked closer at the burdens and resources of clinical psychologists. The manuscript at hand presents different results than the one published by Humer et al. (2023) and Jesser et al. (2024) although using the same sample. The main research questions (RQs) of the current study are as follows:

RQ 1: Qualitative analysis: What was the impact of the COVID-19 pandemic on the work of clinical psychologists?

RQ 2: Qualitative analysis: What support do clinical psychologists wish for during the COVID-19 pandemic?

RQ 3: Mixed-methods analysis: Are there statistically significant differences between different subgroups of clinical psychologists in the frequencies of reporting in the main and subcategories resulting from RQ 1 and RQ 2?

## 2 Materials and methods

### 2.1 Study design

An internet-based cross-sectional survey was conducted between 11 April and 31 May 2022 using Research Electronic Data Capture (REDCap) (Vanderbilt University, Nashville, TN, USA) (Harris et al., 2019). The survey consisted of a total of 49 items. The link to the survey was sent via e-mail to all clinical psychologists registered in the list of the Austrian Federal Ministry of Social Affairs, Health, Care and Consumer Protection (>11,000 clinical psychologists registered in April 2022), provided they had given a valid e-mail address (≈5,000 clinical psychologists). Parallel to this study, a survey on the same topic was conducted among Austrian psychotherapists. As some respondents (*n* = 139) were registered as both clinical psychologists and psychotherapists, only those registered as clinical psychologists (*n* = 172) were used in this study. The results of the survey among psychotherapists are published elsewhere (Winter et al., 2023). Among the ≈ 5,000 clinical psychologists that could be contacted, around 3,000 were not registered as psychotherapists.

The study was conducted after approval by the data protection officer and the ethics committee of the University of Continuing Education Krems, Austria (ethics number: EK GZ 11/2021–2024). All participating clinical psychologists provided electronic consent to participate. The clinical psychologists were not compensated for their time and effort, and participation was voluntary.

### 2.2 Measures

All questions used in the online survey for this study are summarized in Supplementary File 2: Table S1.

#### 2.2.1 Socio-demographic and occupational variables

Data was collected on gender, age, region, and years of employment (the time since the participants were registered in the official list of licensed clinical psychologists). All participants were asked about the type of employment (private practice, outpatient facility, inpatient facility) and whether they received all their income from clinical psychological treatments. They were also asked about the number of patients they provided clinical psychological treatment to on average per week via face-to-face, internet and telephone at the time of the survey. Other occupational variables were the group of patients treated (children and adolescents, adults) and the setting where the treatment occurred (treatment of individuals, partners, families, or groups).

#### 2.2.2 Open-ended questions

To assess the perceived impact of the pandemic on the work of a clinical psychologist, the following open-ended question was asked:

1. What direct or indirect effects did the pandemic have on your work as a clinical psychologist?

A closed and an open-ended question was asked to assess the perceived need for support in working as a clinical psychologist. The questions were as follows:

1. Would you wish support concerning your professional activity as a clinical psychologist? Alternative answer: Yes/No
2. What support would you wish for your professional activity as a clinical psychologist?

Both the questions and the answers were given in German. For the open-ended questions, there were no predefined answer options; respondents were free to describe their experiences in their own words. The answers ranged from one-word answers to whole paragraphs. It was also possible not to fill in the answer field and to skip any of the open-ended questions.

### 2.3 Data analysis

Socio-demographic and work-related data were analyzed descriptively to describe the socio-demographic and occupational characteristics of the sample.

Responses to the open-ended questions were analyzed using conventional qualitative content analysis (Hsieh and Shannon, 2005) to describe the perceived impact of the pandemic on the work of clinical psychologists and the type of support they desire. All the data was first read by the first author to familiarize herself with the material and gain an overview of all the answers. The answers were read word by word several times. In the process, the categories for the open-ended questions were derived inductively, and category definitions, coding rules and example quotations were documented in a codebook. Subsequently, subcategories similar in content were subsumed into more conceptual main categories. In the next step, the data set was coded with the category list using the software ATLAS.ti (ATLAS.ti Scientific Software Development GmbH, 2023). ATLAS.ti facilitates qualitative data management through systematic organization, coding tools to mark and categorize themes, and mapping functions to diagram linkages between coded concepts. This aids researchers in working with and deriving meaning from unstructured data sources. The software aims to provide an integrated suite of tools for qualitative analysis workflows. Since respondents were free to mention several aspects per question, the assignment of more than one category per answer was possible. After the entire data set was coded, all quotations assigned to a category were read again to check for coding errors. Coding errors were corrected, and the category definitions and coding rules were clarified in case of inaccuracies.

In addition to the qualitative content analysis, multivariable logistic regression analyses were performed to determine the odds for psychologists reporting in the respective main or subcategories in relation to the assessed socio-demographic and occupational characteristics. The response within a main- or subcategory (no, yes) was the dependent variable. The socio-demographic and work-related variables functioned as predictors. The following work-related variables were included as predictors: patient group (adults, children and adolescents, both), setting [working with couples (no, yes), working with families (no, yes), working with groups (no, yes)], professional experience (<10 years, 10–19 years, ≥20 years), source of income (additional income, clinical psychological treatment as the only source of income), and form of employment as a clinical psychologist (private practice, institution, both). Socio-demographic variables included in the statistical analyses were: gender (female, male), age (<40 years, 40–49 years, ≥50 years), region (Eastern Austria, Southern Austria, Western Austria). The first subgroup mentioned for each predictor in brackets served as reference group in the statistical model. Adjusted odds ratios (aOR) were assessed and the significance level was set at 0.05. Statistical analyses were conducted using SPSS version 26 (IBM Corp., Armonk, NY, USA).

## 3 Results

### 3.1 Characteristics of the sample

A total of 172 clinical psychologists participated in the study. Participants were 44.90 ± 7.97 years old and 91.9% female (compared to 85.1% in the list of licensed clinical psychologists). They had been in the profession for 13.91 ± 7.72 years (compared to 12.03 ± 6.91 years for all licensed clinical psychologists), and 74.4% worked in private practice only (Table 1).

**Table 1:** Study sample characteristics.

**Gender**	
Female, % (*N*)	91.9 (158)
Male, % (*N*)	8.1 (14)
**Age in years, M (SD)**	**44.9 (7.97)**
**Region**	
Eastern Austria, % (*N*)	44.7 (77)
Southern Austria, % (*N*)	22.7 (39)
Western Austria, % (*N*)	32.6 (56)
**Professional experience in years, *M* (SD)**	**13.9 (7.72)**
**Number of patients treated per week, *M* (SD)**	**14.1 (9.36)**
Number of patients treated in personal contact, % (SD)	85.2 (20.96)
Number of patients treated via the Internet, % (SD)	7.86 (14.72)
Number of patients treated via the telephone, % (SD)	6.93 (14.63)
**Form of employment as clinical psychologist**	
Private practice, % (*N*)	74.4 (128)
Outpatient facility, % (*N*)	37.8 (65)
Inpatient facility, % (*N*)	27.3 (47)
**Income**	
Additional income	41.3 (71)
Only clinical psychology	58.7 (101)
**Setting**	
Individuals	99.4 (171)
Couples	23.3 (40)
Families	22.7 (39)
Groups	28.5 (49)
**Patient group**	
Only adults	32.0 (55)
Only children and adolescents	12.8 (22)
Children, adolescents, and adults	55.2(95)

The Austrian regions were classified according to NUTS 1 (Nomenclature of territorial units for statistics) into three major socio-economic regions (Eastern Austria: Burgenland, Lower Austria, Vienna; Southern Austria: Carinthia, Syria; Western Austria: Upper Austria, Salzburg, Tyrol, Vorarlberg). Bold values are the means of the variables indicated.

### 3.2 Qualitative results

Of the *N* = 172, 86.0% (*n* = 148) answered the first question about the impact of the pandemic on their work as a clinical psychologist. 41.9% (*n* = 72) answered YES to the closed question about the desire for support related to their professional work as a clinical psychologist, and 58.1% (*n* = 100) answered NO. 40.1% (*n* = 69) responded to the open-ended question about the support they would like in relation to their professional activity as a clinical psychologist.

Qualitative content analysis resulted in five main categories and 16 subcategories related to the question of the impact of the pandemic on the work of clinical psychologists (Question 1). Three main categories and ten subcategories were formed to address the psychologists’ wish for support (Question 3). All main and subcategories are shown in Tables 2, 3.

**Table 2:** Percentage of respondents reporting each main category (in bold) and subcategory of changes in their work as a clinical psychologist in Austria due to the COVID-19 pandemic.

**Categories**	***n***	**%**
**Impact of the pandemic on the number of patients**	**75**	**43.6%**
Increase in the number of patients	43	25.0%
Decrease in the number of patients	38	22.1%
**Impact of the pandemic on the treatment setting**	**70**	**40.7%**
Voice and video calls	44	25.6%
Wearing a mask	25	14.5%
Other COVID-19 measures	25	14.5%
**Impact of the pandemic on working conditions**	**58**	**33.7%**
Workload and work effort	41	23.8%
Financial impact on psychologists	11	6.4%
Collaboration and working atmosphere	10	5.8%
Uncertainty and insecurity about COVID-19 regulations	6	3.5%
More flexibility and availability	5	2.9%
**Impact of the pandemic on work with patients**	**39**	**22.7%**
Relationship and communication	23	13.4%
Focus of treatment	13	7.6%
Financial situation of patients	7	4.1%
**Impact of the pandemic on patients’ mental health**	**35**	**20.3%**
Crises and deterioration in mental health	19	11.0%
Diversity of stressful feelings	16	9.3%
Diversity of disorders	14	8.1%

Question 1: “What direct or indirect effect did the pandemic have on your work as a clinical psychologist?.” The percentages of the main categories may differ from the sum of the percentages in the individual subcategories because it may be that a respondent reported experiences in several subcategories (e.g., financial situation of patients and focus of treatment) within one main category (e.g., impact of the pandemic on work with patients).

**Table 3:** Percentages of respondents reporting each main category (in bold) and subcategory of support wishes.

**Categories**	***n***	**%**
**Political and legislative support**	**34**	**19.8%**
Psychological treatment as a statutory service	27	15.7%
Increase in staff within institutions	9	5.2%
Professional associations	2	1.2%
**Networking and sharing of information**	**31**	**18.0%**
Supervision and intervision	17	9.9%
Team exchange and networking	10	5.8%
Training and education online	5	2.9%
Guidelines and measures	5	2.9%
**Improvement of working conditions**	**28**	**16.3%**
Human and time resources	13	7.6%
Financial support for clinical psychologists	11	6.4%
Appreciation and recognition	7	4.0%

Question 2: “What support concerning your professional activity as a clinical psychologist would you wish for?.” The percentages of the main categories may differ from the sum of the percentages in the individual subcategories because it may be that a respondent reported support wishes in several subcategories (e.g., human and time resources and financial support for clinical psychologists) within one main category (e.g., improvement of working conditions).

#### 3.2.1 Results for RQ 1: impact of the pandemic on work as a clinical psychologist

Main category 1: The largest main category, mentioned by 43.6% of clinical psychologists, is related to the change in the number of patients during the pandemic. This main category was divided into two subcategories. While 25.0% of participants reported an increase in patient numbers, 22.1% reported a loss of patients and a decrease in demand. It was noted that there was a decrease in demand, particularly at the beginning of the pandemic. However, over time, there was an increase in demand for clinical psychological treatment, particularly in private practice. Some clinical psychologists reported an increased need for clinical psychological treatment among children and adolescents. In inpatient and/or group settings, on the other hand, patients terminated treatment due to the COVID-19 measures. Increased cancellations and postponements of appointments were also mentioned. One participant (705) observed: “More cancellations at short notice, due to a positive COVID-19 test or contact person number one (K1) quarantine and lockdown.” Participants stated that patients were less reliable during the pandemic, making scheduling difficult.

Main category 2: The second most frequently mentioned main category, reported by 40.7% of respondents, was a change in the treatment setting. The main category comprises three subcategories. 25.6% of clinical psychologists reported a change to voice or video calls. Most statements within this subcategory conveyed a neutral attitude towards remote treatment. However, both advantages and disadvantages were mentioned. For example, one person (465) said: “More work via Zoom and phone - an advantage is that it is possible everywhere, a disadvantage is that I have to be more attentive otherwise details are lost more easily (especially body language).” Clinical psychologists also noted that in some cases, the introduction of voice and video calls was not well received by their patients, who preferred to continue with face-to-face treatment. For example, one clinical psychologist (626) stated: “My patients have reported being grateful to be able to return to the practice in person under safe circumstances.”

A further 14.5% of clinical psychologists mentioned wearing a mask as one of the effects of the pandemic on their work. Participants described wearing the mask as physically demanding and a barrier to building good relationships because of the loss of facial information. For example, one person (381) said: “Masks are annoying and create distance in conversations”; another participant (656) said: “Wearing a mask was sometimes distracting, e.g., when working out emotions.”

The use of disinfectants, air purification, wearing protective clothing, and increasing distance from patients, home office and outdoor settings were mentioned by 14.5% of respondents as additional pandemic containment strategies that directly affected the work of clinical psychologists.

Main category 3: 33.7% of clinical psychologists reported an impact of the pandemic on working conditions in both private practice and institutional settings. There were five subcategories within this main category. 23.8% of respondents noted an increased workload due to pandemic-related measures and changes - and thus an increased burden. The pandemic resulted in additional administrative and bureaucratic work and time pressure, leading to feelings of strain and overwork. One person (Berufsverband Österreicher PsychologInnen, 2023) expressed: “Work more exhausting, confrontation with resignation.” Another person (491) observed: “High work pressure, frequent case requests, quick appointments are expected.” For another clinical psychologist, compliance with COVID-19 measures was associated with an increased workload. The fact that some patients did not want to comply with the measures was also perceived as a burden. Participants also discussed fear of infection at work.

6.4% of clinical psychologists reported experiencing a financial impact from the pandemic. Respondents experienced a loss of income, particularly at the beginning of the pandemic. One person (633) noted: “I opened my practice on 15 March 2020 (lockdown started on 16 March), i.e., at the beginning I only had costs and no income.” Clinical psychologists in the institutional sector stated that they had to accept a reduction in working hours and, thus, a reduction in their salary.

A further 5.8% of clinical psychologists said they missed the exchange with their colleagues as the COVID-19 measures limited team communication. The working atmosphere was described as tense. One participant (291) reported: “The working atmosphere is much more determined by guidelines and collegial stress.”

3.5% of clinical psychologists reported feeling insecure in the workplace due to repeated changes in COVID-19 regulations. In particular, they raised the issue of uncertainty and insecurity about how long new regulations and policies would be in place and how they would be managed. One participant (90) reported: “Uncertainty, anxiety about how to deal with the measures in practice.”

Another impact of the pandemic on working conditions was the need for more flexible availability (2.9%). One participant (373) reported: “Shifts due to quarantine of clients and their relatives required more flexibility than usual.” Positive aspects of flexible working were also mentioned. One respondent (465) commented that the spatial flexibility provided by remote treatment was an advantage at work.

Main category 4: Another main category, mentioned by 22.7% of respondents, relates to the impact of the pandemic on their work with patients. The main category was divided into three subcategories. 13.4% of clinical psychologists experienced difficulties establishing and maintaining good patient relationships. Reasons given included being unable to meet in person, wearing the mask, and introducing voice and video telephony into treatment. Clinical psychologists reported difficulties in communicating with their patients. One participant stated (281): “By wearing FFP2 masks, an important part of facial expression is lost, and this creates a feeling of not knowing the other person.” Another participant (691) described: “Many topics were lost because telephone conversations were much shorter, more superficial and less psychological.”

7.6% of clinical psychologists experienced a shift in the focus of treatment. They found that pandemic-related issues dominated their conversations with patients. As a result, other matters were pushed into the background. Stabilization, resilience and increasing patients’ resources were identified as the focus of psychological treatment during the pandemic. One participant (489) stated: “The pandemic took up a lot of space in therapy that was meant for the clients’ problems. The reduction in face-to-face contact and the use of the mask meant that clinical psychologists experienced more distance or less closeness between themselves and their patients, thus reducing the depth of treatment. they explained this, among other things, by the lack of opportunity to use psychological interventions on the phone and in video telephony or by the fact that the focus of the conversations was on COVID-19. One person (531) stated: “individual methods difficult to impossible to implement by telephone/online, therefore reduced variety of methods/offers; confidence-building measures for individual persons reduced, therefore less in-depth.”

Clinical psychologists also reported on the financial situation of their patients (4.1%). They stated that the pandemic had increased the need for psychological treatment but also worsened the financial situation of their patients. One person (200) reported: “Many requests from patients who cannot afford the treatment.”

Main category 5: Another main category mentioned by 20.3% of respondents was the perceived impact of the pandemic on patients’ mental health. This category was divided into three subcategories. 11.0% of clinical psychologists reported a deterioration in mental health. They observed an increase in the severity of symptoms and a more pronounced manifestation of symptoms, combined with bottlenecks in the admission of the patients to inpatient psychiatric care. For example, some respondents observed that patients had more varied, severe, and chronic symptoms. Crises in treatment were also more frequent during the pandemic. Another aspect related to the mental state of patients was the variety of distressing feelings mentioned by 9.3% of respondents; for example, insecurity and anxiety, withdrawal and loneliness were more frequently observed in patients. Finally, 8.1% of clinical psychologists specifically mentioned certain disorders they had seen more often than usual during the pandemic, including depression, impulse control disorders, eating disorders, post-COVID syndrome and anxiety disorders. One (245) observed: “Depression, eating disorders, compulsions, anxiety in children and adolescents.”

#### 3.2.2 Results for RQ 2: wishes for support in clinical psychological work

Main category 1: The most frequently mentioned main category was the wish for support at a political and legislative level (19.8%). This main category was divided into three subcategories. 15.7% of clinical psychologists said they wanted to see clinical psychological treatment as a statutory service. 5.2% of clinical psychologists would like to see an expansion of institutional capacity and increase in staff in institutions. Respondents cited the need to expand outpatient and inpatient services for children and adolescents. For example, one respondent (Sevecke et al., 2023) commented: “URGENT: expansion of child and adolescent psychiatry!!!!!!!!! And more services there. Capacities of all counselling centers should be increased [...].”1.2% of clinical psychologists wanted support from professional associations.

Main category 2: Another main category, mentioned by 18% of respondents, addresses the desire to network and share information. This main category was divided into four subcategories. 9.9% of clinical psychologists wished for more supervision and intervision during the pandemic. 5.8% of the participants wished for more team exchange within institutions or more networking and meetings between colleagues working in private practice. 2.9% of clinical psychologists wished for more online training and further education on topics related to the pandemic, for example, long-COVID. Finally, 2.9% of clinical psychologists wished for clear communication of guidelines and measures to prevent the spread of COVID-19. One person (373) reported: “Precise differentiation of measures for the practice and the institutional context.”

Main category 3: “Improving working conditions’ is another main category, mentioned by 16.3%. It is divided into three subcategories. 7.6% of respondents wanted support in terms of human and time resources. They hoped to feel less time pressure in doing their work. Clinical psychologists working in private practice indicated they would like more support in planning, organizing, and managing their work, as well as staff to help with it. Clinical psychologists reported that they would like more time off and holidays. A further 6.4% of clinical psychologists wanted financial support for training, supervision, and self-care. They also wanted to be paid more for their work and generally felt that social and health professionals should earn more. Finally, 4.0% of clinical psychologists said they would like more appreciation and recognition for their work. One respondent (732) stated: “Social recognition, systemic recognition.”

### 3.3 Results for RQ 3: mixed-methods analysis

#### 3.3.1 Impact of the pandemic on the work of clinical psychologists

Working with different *patient groups* (adults, children, and adolescents) was associated with the odds for reporting effects of the pandemic on collaboration and working atmosphere. Clinical psychologists working with children and adolescents were more likely to report differences in this subcategory compared to those working solely with adults (children and adolescents vs. solely adults: aOR: 23.79, *p* = 0.043; children, adolescents, and adults vs. solely adults: aOR: 29.34, *p* = 0.021). Also, the likelihood of reporting a higher diversity of stressful feelings in their patients due to the pandemic was higher in clinical psychologists working with children and adolescents compared to those working exclusively with adults (aOR: 7.30, *p* = 0.045).

The *setting* (partners, families, groups) was associated with the likelihood for reporting an impact of the pandemic on the work with patients. Clinical psychologist working with families compared to those providing no treatments to families were less likely to report in this main category (aOR: 0.249, *p* = 0.026).

Significant findings concerning *professional experience* were observed for the likelihood of clinical psychologists reporting an impact of the pandemic on changes in the number of patients. Clinical psychologist with 10–19 years in the profession and those with at least 20 years in the profession had higher odds for reporting changes in the patient number than those with less than 10 years in the profession (10–19 years vs. <10 years, aOR: 4.24, *p* = 0.003; ≥20 years vs. <10 years: aOR: 5.34, *p* = 0.009). Similarly, being longer in the profession increased the likelihood of reporting an increase in patient numbers (10–19 years vs. <10 years, aOR: 4.81, *p* = 0.009; ≥20 years vs. <10 years: aOR: 6.53, *p* = 0.015).

Several differences related to the *source of income* were identified. Clinical psychologists relying solely on clinical psychological treatment as their source of income were more likely reported on a decrease in the number of patients than those with additional income sources (aOR: 2.53, *p* = 0.044). Furthermore, they had a lower chance to report about other COVID-19 measures such as home office (aOR: 0.348, *p* = 0.036). Clinical psychologists relying solely on clinical psychological treatment as their source of income more likely reported changes in their working conditions due to the pandemic than those with additional income sources (aOR: 2.23, *p* = 0.041).

Differences according to the *form of employment* as clinical psychologists were found in the subcategory *collaboration and working atmosphere*, with clinical psychologists working in an institution showing higher odds for reporting changes in collaboration due to the pandemic than clinical psychologists working in private practice (aOR: 10.6, *p* = 0.043).

Considering all assessed socio-demographic and work-related variables simultaneously in the statistical model revealed no differences in the likelihood of clinical psychologists reporting on the impact of the pandemic on their work regarding age.

The odds for reporting changes in collaboration and working atmosphere were higher in male then female clinical psychologists (aOR: 10.61, *p* = 0.043).

The likelihood for reporting an impact of the pandemic on treatment setting was higher in Western Austria compared to Eastern Austria (aOR: 2.29, *p* = 0.032). Similarly, clinical psychologist from Western Austria compared to Eastern Austria had higher odds for reporting an impact of the pandemic on the work with patients (aOR: 2.72, *p* = 0.030). Changes in collaboration and working atmosphere were less likely to be reported by clinical psychologists from Western Austria (aOR: 0.060, *p* = 0.034) and Southern Austria (aOR: 0.069, *p* = 0.043) compared to Eastern Austria.

#### 3.3.2 Support wishes of clinical psychologists

Differences in support wishes were found in the closed-ended question on the general *wish for support* (yes vs. no) regarding the *patient groups* the clinical psychologists were working with. Clinical psychologists working with children and adolescents were more likely to report that they wanted support compared to those working solely with adults (aOR: 5.71, *p* = 0.005).

The *setting* (partners, families, groups) was associated with some differences. Clinical psychologists working with families were more likely to express a wish for support compared to those providing no treatments to families (aOR: 2.94, *p* = 0.017). These psychologists were also more likely to wish for more human and time resources compared to those not working with families (aOR: 8.33, *p* = 0.008). Clinical psychologists treating groups had higher odds for wishing more appreciation and recognition than those not working with groups (aOR: 11.26, *p* = 0.032).

No difference in the support wishes regarding what type of support they desired was found with respect to *professional experience*, *source of income,* and *occupational status*.

Among the socio-demographic variables, no differences were observed with respect to *gender* and *region*, while *age* differences were observed for the likelihood to wish for more networking and sharing of information. More specifically, the likelihood for wishing more exchange was higher in clinical psychologists aged 40–49 (aOR: 6.43, *p* = 0.016) and ≥50 years (aOR: 6.05, *p* = 0.025) compared to those younger than 40 years.

## 4 Discussion

### 4.1 Impact of the pandemic on the work of clinical psychologists – patient numbers and deterioration of mental health

This study showed that clinical psychologists perceived changes in patient numbers and treatment settings as the most significant changes caused by the COVID-19 pandemic. Concerning patient numbers, it is crucial to consider a temporal aspect and distinguish the beginning from the later stages of the pandemic. Clinical psychologists found that the number of patients decreased at the beginning of the pandemic in spring 2020. Patients often cancelled appointments at short notice and were not always reliable. After the first lockdown in May 2020, clinical psychologists noticed a deterioration in their patients’ mental health and an increase in their need for clinical psychological treatment. These findings are consistent with the results of the study by Winter et al. (2023). In their study, after a decrease in the total number of patients treated in the first weeks of the pandemic, an increase in the number of patients was observed in the second and third year of the pandemic. In the study by Winter et al. (2023), the number of patients even exceeded pre-pandemic levels. In the current study, clinical psychologists with more professional experience were particularly in demand. They may have a more extensive network of contacts through years of practice or may have better referral channels.

In our study, clinical psychologists reported that their patients’ mental health deteriorated throughout the pandemic, with more frequent crises and worsening symptoms, which is consistent with the international research literature (Nochaiwong et al., 2021; Leung et al., 2022; Mahmud et al., 2023). However, it would be short-sighted to attribute the deterioration in mental health solely to the effects of the pandemic and its measures. In 2022, new geopolitical crises such as the war in Ukraine, the energy crisis and related inflation came to the fore. In a qualitative study, Gächter et al. compared the concerns of a representative sample of the Austrian population at two different points in time (winter 2020/21 and spring 2022) (Gächter et al., 2023). In 2020/21, the COVID-19 restrictions and their effects were the primary concerns. In 2022, the main concerns were inflation, finances, and the war in Ukraine. At both points in time, concerns about mental health remained high. In a survey conducted in Austria in June 2022 on fears about the consequences of the Russian-Ukrainian war, a total of 92% of respondents were very worried about higher food prices (Mohr, 2022). This was the greatest fear associated with the conflict, followed by the fear of higher energy prices, reported by 90% (Mohr, 2022).

### 4.2 Impact of the pandemic on the work of clinical psychologists – bottlenecks in the treatment of children and adolescents

The increase in demand for clinical psychological treatment was particularly marked among children and adolescents. Respondents working with children and adolescents reported a higher diversity of stressful feelings in their patients. Compared to their colleagues treating adult patients, psychologists working with children and adolescents more frequently reported changes in collaboration and working atmosphere, which may indicate an increased need for networking with other professionals and institutions in order to provide the necessary care for their patients, as well as exhaustion of psychologists due to the effort and difficult conditions, as also found by Fiala-Baumann et al. (2022). Respondents in our study were also critical of the lack of inpatient psychiatric care. These results confirm findings in the literature that children and adolescents were the group most affected by the negative psychological effects of the COVID-19 pandemic and the accompanying containment measures (Racine et al., 2021). This was also supported by findings from Austria (Pieh et al., 2021; Sevecke et al., 2023). It seems quite conclusive that in our study, clinical psychologists working with children and adolescents as well as those working with families were more likely to say that they would appreciate support in their work. Psychosocial care for children and adolescents in Austria has received increasing attention in recent years. Due to the challenges of the COVID-19 pandemic, it has become clear that there is a need for a rapid expansion of child and adolescent mental health care as well as low-threshold and low-cost or free psychological and psychotherapeutic services (Culen et al., 2020).

### 4.3 Support needs – calls for broader coverage of treatment costs

When asked what support they would like to see, clinical psychologists in our study also named political and legislative support. In particular they stressed the need for clinical psychological services to be covered by health insurance. This demand is reflected in the results of Winter et al.’s study of psychotherapists in Austria (Winter et al., 2023). Psychotherapists also advocated for low-cost psychotherapy and less bureaucracy in reimbursing treatment costs. Given the overall deterioration in patients’ mental health and the observation by clinical psychologists that some patients are struggling with the financial consequences of the pandemic, it seems important from a health policy perspective to take steps in this area. At the time the survey was conducted, clinical psychological treatment costs were not covered by health insurance funds. However, it was deemed a significant achievement when, in July 2023, clinical psychological treatment was included in the benefit catalogue of the General Social Insurance Act (ASVG) (Berufsverband Österreicher PsychologInnen, 2023). Participants in our study would like to see a similar increase in resources for inpatient psychiatric care.

### 4.4 Impact of the pandemic on the work of clinical psychologists – introduction of remote treatment formats

In terms of changes in the treatment setting, clinical psychologists in our study described a shift towards remote treatment. The effectiveness of clinical psychological or psychotherapeutic treatment via videoconferencing has been extensively studied prior to the pandemic for specific psychotherapeutic approaches, such as cognitive behavioral therapy or interpersonal psychotherapy (Mohr et al., 2012; Carlbring et al., 2018). Several studies have shown efficacy for most clinical conditions, including anxiety, depression, and post-traumatic disorder (Mohr et al., 2008; Cuijpers et al., 2019). In addition, studies have found no difference between face-to-face and online treatment in terms of the quality of the psychotherapeutic relationship (Sucala et al., 2012; Poletti et al., 2021). The implementation of videoconferencing-based healthcare delivery during the COVID-19 pandemic yielded comparable clinical outcomes to traditional in-person care modalities. These real-world observations substantiate the findings of prior randomized controlled trials and meta-analytic investigations, wherein direct comparisons were made between videoconferencing and in-person care approaches concerning their respective clinical efficacies (Beurs et al., 2022). Other research conducted during the pandemic has already shown that the COVID-19 pandemic has prompted psychologists and other mental health professionals to increase their use of web-based applications, particularly videoconferencing tools (Swartz, 2020; Markowitz et al., 2021). Winter et al. (2023) showed that the highest proportion of internet-based psychotherapeutic treatments in Austria was observed during the lockdown period in 2020. Although it decreased significantly as the pandemic progressed, it was still higher in 2022 than before the pandemic. Other studies have confirmed that Austrian psychotherapists continued to use internet-based treatment even after the lockdown restrictions were lifted and it was legally possible to offer face-to-face treatment again (Höfner et al., 2021; Stefan et al., 2021; Stadler et al., 2023). Psychotherapists likely came to appreciate the benefits of online interventions, such as spatial flexibility, reduced travel time and costs, and access for patients who could not otherwise travel to the practice due to long distances (Stefan et al., 2021). However, the willingness of therapists to use remote treatment formats depends on several factors, including the therapist’s familiarity with the tools needed for online treatment and the ability of the patient and therapist to adapt to the online format (Höfner et al., 2021). In the current study, clinical psychologists confirmed the importance of online treatment in reaching groups of patients who would not otherwise have the opportunity to receive clinical psychological treatment. They also cited increased spatial and temporal flexibility as an advantage of virtual clinical psychological treatment. At the time of the survey in the spring of 2022, just under 15% of patients received clinical psychological treatment via telephone or the internet.

### 4.5 Impact of the pandemic on the work of clinical psychologists – burdens caused by COVID-19 measures

Other experiences made by the clinical psychologists in our study were perceived as more distressing. For example, wearing masks caused communication difficulties, and respondents described exhaustion and fatigue after several hours of mask use at work. Compliance with COVID-19 containment measures, such as wearing protective clothing, keeping a distance, or using air purifiers, was considered an additional burden. Other studies showed similar results for healthcare workers. Prolonged use of filtering respirators can lead to increased symptoms of exertion, shortness of breath, headache, fatigue, and difficulty communicating, as well as depressive and anxiety symptoms (Cigiloglu et al., 2022; Sahebi et al., 2022). Our study also showed that the pandemic also resulted in increased administrative work. For example, treatment appointments had to be rescheduled, and information on current measures had to be obtained. In addition, working conditions became more tense with less opportunity for team or colleague interaction, which was particularly mentioned by clinical psychologists working in an institutional setting. While clinical psychologists working in private practice probably had more freedom to decide how to manage their day-to-day work, those working in an institution had to follow a strict COVID-19 protocol. In addition, most institutional settings were run at maximum capacity, whereas clinical psychologists in private practice could decide how many patients they wanted to see. Other research findings support the hypothesis that the institutional setting was also perceived as more stressful than private practice by other mental health professionals during the pandemic. Schaffler et al.’s study of psychotherapists (Schaffler et al., 2023) showed that those working in an institutional setting reported poorer well-being.

### 4.6 Support needs – provision of structures for work organization and collaboration in times of crisis

The findings described in the previous section highlight the importance of improving working conditions and providing workplace support for clinical psychologists to meet the additional demands of the pandemic. The different needs of clinical psychologists in employment and in private practice should be taken into account. Finding ways to meet clinical psychologists’ needs for more opportunities for regeneration and more team interaction (communication within teams and with colleagues, intervision, and supervision) could help to maintain the resilience and capacity of this professional group in the long term, even in times of crisis. A significant challenge is finding solutions for clinical psychologists working in outpatient settings without a secure income.

While more opportunities for networking and communication with colleagues and improvements in working conditions were also areas of support mentioned by psychotherapists in the Winter et al. study (Winter et al., 2023), skills development and training, which was mentioned by a large proportion of psychotherapists, played a minor role for clinical psychologists. It is possible that training as a clinical psychologist provides better preparation to deal with acute social and health crises or to adapt to settings other than face-to-face. The differentiation of psychotherapy training into many different psychotherapy methods (23 different psychotherapy methods are recognized in Austria) (Heidegger, 2017) may mean a loss in teaching a broader range of interventions and techniques in treating patients in a state of crisis.

There exist a number of analogous findings between the psychotherapists studied by Winter et al. (2023) and the current examination’s sample of clinical psychologists. While the two studies have some parallels in terms of the observed impact of the pandemic and the desired areas of support, there are also notable differences between the outcomes of the clinical psychologist group compared to the psychotherapists.

Both studies found an initial drop in patient volume at the beginning of the pandemic, followed by a rebound over time. Winter et al. showed volumes exceeding pre-pandemic levels over time. Clinical psychologists and psychotherapists surveyed identified similar needs for more networking/communication opportunities and improvements in working conditions. Both groups expressed a desire for more political/legislative support, such as coverage of psychological services by health insurance. The current study highlighted that clinical psychologists underlined the value of online treatments to access more patients. Winter et al. also found similar results regarding the importance of online treatments. The desire for skills development regarding crisis coping due to the pandemic played a more important role for the psychotherapists surveyed by Winter et al. than for the clinical psychologists in the current study, possibly due to differences in crisis preparedness in their training. The current study found a higher demand for clinical psychologists with more professional experience, while Winter et al. did not identify differences based on years of practice.

Mental disorders are widespread in Austria and cause high personal, social, and financial costs (Löffler-Stastka and Hochgerner, 2021). Clinical psychologists, together with psychotherapists, provide the majority of clinical treatment for patients with mental health problems (Löffler-Stastka and Hochgerner, 2021). They also provide help in acute crises, offer counselling, and cooperate with other health professionals. This study highlighted the changes that clinical psychologists experienced during the pandemic and the support wishes they identified for their profession. In view of comparable findings on psychological distress in other countries (Budimir et al., 2021), which indicate a similar shortage of care (Bundes Psychotherapeuten Kammer, 2023), we conclude that the results of our study are also relevant for other national contexts. Expanding psychosocial services, covered by health insurance, and supporting professionals in the field through suitable legal structures, administrative help, financial resources, and networking and training platforms can establish a vital groundwork for effectively coping with future crises.

## 5 Limitations

This study has several limitations. Firstly, the study was conducted using an online questionnaire, which may have led to a bias towards people who are open to electronic data processing and the use of online tools. Another limitation in analyzing the results is that there is no data on the situation before the COVID-19 pandemic to compare the results with. In addition, recall bias is possible, as participants reported on situations from the start of the pandemic in 2020 until the time of the survey in spring 2022. It is also crucial to acknowledge the inherent limitation of the cross-sectional design of the study, which precludes causal inference. The current sample represents a relatively selective subgroup of the ≈ 3,000 eligible participants. We suspect a response rate around 6%, which is considered quite low. Such a limited response poses an augmented risk of response bias, whereby those with intense or polarized perspectives on the matter disproportionately opted to provide input. Finally, it should be noted that we had no data from patients, only information from clinical psychologists.

## 6 Conclusion

The results of this study highlighted the importance of mental health services in times of pandemic and crisis. At the same time, the results again showed that participants still perceive significant gaps in the mental health care system, and that they experience that financially disadvantaged patients, children and adolescents in particular, have difficulties in accessing clinical psychological services. At the level of the health care system, clinical psychologists expressed a need for more fully or partially funded contingents for clinical psychological treatment and an increase in beds and staff in inpatient psychiatric care. At the level of professional policy, participants see a need for support for the profession of clinical psychology. This could take the form of assistance in coping with additional organizational and administrative tasks, more opportunities for exchange within teams or between colleagues, or financial support in the event of a loss of income.

## Data availability statement

The raw data supporting the conclusions of this article will be made available by the authors, without undue reservation.

## Ethics statement

The studies involving humans were approved by Ethics committee of the University of Continuing Education Krems, Austria. The studies were conducted in accordance with the local legislation and institutional requirements. The participants provided their written informed consent to participate in this study.

## Author contributions

PS: Formal analysis, Visualization, Writing – original draft. EH: Conceptualization, Data curation, Formal analysis, Investigation, Methodology, Project administration, Software, Supervision, Validation, Visualization, Writing – original draft. YS: Conceptualization, Methodology, Writing – review & editing. CP: Conceptualization, Writing – review & editing. TP: Writing – review & editing. AF: Writing – review & editing. OK: Writing – review & editing. IN: Writing – review & editing. AJ: Conceptualization, Formal analysis, Methodology, Supervision, Writing – original draft.
