# Supplementary material for: Impact of the COVID-19 pandemic on the work of clinical psychologists in Austria: results of a mixed-methods study
Source: Front Psychol. 2024 Apr 25;15:1302442. doi: 10.3389/fpsyg.2024.1302442 (PMC11081068; doi:10.3389/fpsyg.2024.1302442)
Supplement: Supplementary file 1 [file Table_1.DOCX]

Supplementary Material

**Impact of the COVID-19 pandemic on the work of clinical psychologists in Austria: results of a mixed methods study**

# Supplementary Figures and Tables

| **Suppl. Table S1** | |
| --- | --- |
| **Study sample characteristics** | |
| **Gender** | |
| Female, % (N) | 91.9 (158) |
| Male, % (N) | 8.1 (14) |
| **Age in years, M (SD)** | **44.9 (7.97)** |
| **Region** | |
| Eastern Austria, % (N) | 44.7 (77) |
| Southern Austria, % (N) | 22.7 (39) |
| Western Austria, % (N) | 32.6 (56) |
| **Professional experience in years, M (SD)** | **13.9 (7.72)** |
| **Number of patients treated per week, M (SD)** | **14.1 (9.36)** |
| Proportion of patients treated in personal contact, % (SD) | 85.2 (20.96) |
| Proportion of patients treated via the Internet, % (SD) | 7.86 (14.72) |
| Proportion of patients treated via the telephone, % (SD) | 6.93 (14.63) |
| **Form of employment as clinical psychologist** | |
| Private practice, % (N) | 74.4 (128) |
| Outpatient facility, % (N) | 37.8 (65) |
| Inpatient facility, %(N) | 27.3 (47) |
| **Income** | |
| Only clinical psychology | 41.3 (71) |
| Additional income | 58.7 (101) |
| **Setting** | |
| Individuals | 99.4 (171) |
| Couples | 23.3 (40) |
| Families | 22.7 (39) |
| Groups | 28.5 (49) |
| **Patient group** |  |
| Only adults | 32.0 (55) |
| Only children and adolescents | 12.8 (22) |
| Children, adolescents, and adults | 55.2(95) |

**Suppl. Table S2.** Percentage of respondents reporting each main category (in bold) and subcategory of changes in their work as a clinical psychologists in Austria due to the COVID-19 pandemic. Question 1: "What direct or indirect effect did the pandemic have on your work as a clinical psychologist?".

| **Categories** | ***n*** | **%** |
| --- | --- | --- |
| **Impact of the pandemic on the number of patients** | **75** | **43.6%** |
| Increase in the number of patients | 43 | 25.0% |
| Decrease in the number of patients | 38 | 22.1% |
| **Impact of the pandemic on the treatment setting** | **70** | **40.7%** |
| Voice and Video calls | 44 | 25.6% |
| Wearing a mask | 25 | 14.5% |
| Other COVID-19 measures | 25 | 14.5% |
| **Impact of the pandemic on working conditions** | **58** | **33.7%** |
| Workload and work effort | 41 | 23.8% |
| Financial impact on psychologists | 11 | 6.4% |
| Collaboration and working atmosphere | 10 | 5.8% |
| Uncertainty and insecurity about Covid-19 regulations | 6 | 3.5% |
| More flexibility and availability | 5 | 2.9% |
| **Impact of the pandemic on work with patients** | **39** | **22.7%** |
| Relationship and communication | 23 | 13.4% |
| Focus of treatment | 13 | 7.6% |
| Financial situation of patients | 7 | 4.1% |
| **Impact of the pandemic on patients' mental health** | **35** | **20.3%** |
| Crises and deterioration in mental health | 19 | 11.0% |
| Diversity of stressful feelings | 16 | 9.3% |
| Diversity of disorders | 14 | 8.1% |

*Note:* The percentages of the main categories may differ from the sum of the percentages in the individual subcategories because it may be that a respondent reported experiences in several subcategories (e.g., financial situation of patients and focus of treatment) within one main category (e.g., impact of the pandemic on work with patients).

**Suppl. Table S3.** Percentages of respondents reporting each main category (in bold) and subcategory of support wishes. Question 2: "What support concerning your professional activity as a clinical psychologist would you wish for?".

| **Categories** | ***n*** | **%** |
| --- | --- | --- |
| **Political and legislative support** | **34** | **19.8%** |
| Psychological treatment as a statutory service | 27 | 15.6% |
| Increase in staff within institutions | 9 | 5.2% |
| Professional associations | 2 | 1.2% |
| **Networking and sharing of information** | **31** | **18.0%** |
| Supervision and intervision | 17 | 9.9% |
| Team exchange and networking | 10 | 5.8% |
| Training and education online | 5 | 2.9% |
| Guidelines and measures | 5 | 2.9% |
| **Improvement of working conditions** | **28** | **16.3%** |
| Human and time resources | 13 | 7.6% |
| Financial support for clinical psychologists | 11 | 6.4% |
| Appreciation and recognition | 7 | 4.0% |

*Note:* The percentages of the main categories may differ from the sum of the percentages in the individual subcategories because it may be that a respondent reported support wishes in several subcategories (e.g., human and time resources and financial support for clinical psychologists) within one main category (e.g., improvement of working conditions).
