# Supplementary material for: Impact of the COVID-19 pandemic on the work of clinical psychologists in Austria: results of a mixed-methods study
Source: Front Psychol. 2024 Apr 25;15:1302442. doi: 10.3389/fpsyg.2024.1302442 (PMC11081068; doi:10.3389/fpsyg.2024.1302442)
Supplement: Supplementary file 2 [file Table_2.DOCX]

Supplementary Material

**Impact of the COVID-19 pandemic on the work of clinical psychologists in Austria: Results of a mixed- methods study**

| **Suppl. Table S1: Questions from the online survey translated into English**  Are you a trained clinical psychologist?  □ Yes  □ No  How many patients do you currently treat on average per week in face-to-face clinical psychology sessions? (Please enter a number; choose 0 for no patients) _____  How many patients do you currently treat on average per week in clinical psychology sessions over the internet? (Please enter a number; choose 0 for no patients) _____  How many patients do you currently treat on average per week in clinical psychology sessions over the phone? (Please enter a number; choose 0 for no patients) _____  What direct or indirect effects did the pandemic have on your work as a clinical psychologist?  ____________________________________________________________________________________________________________________________________________________________________________________________________________________________________________________________________________________  Would you wish support concerning your professional activity as a clinical psychologist?  □ Yes  □ No  What support would you wish for your professional activity as a clinical psychologist?  ________________________________________________________________________________________________________________________________________________________________________________________ ____________________________________________________________________________________________  Please answer a few general questions about yourself and your professional activities for statistical purposes.  Please indicate your gender:  □ Female  □ Male  □ Diverse  Please provide your age in years (e.g., 44). _____  Please specify the federal state in which you are professionally active:  □ Upper Austria  □ Lower Austria  □ Vienna  □ Styria  □ Burgenland  □ Carinthia  □ Salzburg  □ Tyrol  □ Vorarlberg  When were you registered in the list of clinical psychologists by the Ministry? Please enter only the year "yyyy" (e.g., 2019). Tip: If you are unsure, you can easily check the year of your registration at the following link: <http://klinischepsychologie.ehealth.gv.at/>  _______  Do you treat adult patients and/or children or adolescents clinically?  □ Adults  □ Children/Adolescents  □ Adults and Children/Adolescents  In which setting(s) do you work clinically?  (Multiple selections are possible)  □ Individual therapy  □ Couple therapy  □ Family therapy  □ Group therapy  Please specify the context in which you work clinically.  (Multiple selections are possible)  □ Private practice  □ Outpatient institution  □ Inpatient institution  Are your clinical psychology treatments currently your only sources of income?  □ Yes  □ No |
| --- |
